# Employment outcomes in family supporters of patients with early stage breast cancer and their association with patients’ health‐related quality of life and financial burden

**DOI:** 10.1002/cam4.4513

**Published:** 2022-02-03

**Authors:** Christine M. Veenstra, Thomas M. Braun, Paul H. Abrahamse, Daniela Wittmann, Sarah T. Hawley

**Affiliations:** ^1^ Division of Hematology/Oncology Department of Internal Medicine University of Michigan Ann Arbor Michigan USA; ^2^ Institute for Healthcare Policy and Innovation University of Michigan Ann Arbor Michigan USA; ^3^ Department of Biostatistics University of Michigan Ann Arbor Michigan USA; ^4^ Department of Urology University of Michigan Ann Arbor Michigan USA; ^5^ Department of Internal Medicine University of Michigan Ann Arbor Michigan USA

**Keywords:** breast cancer, caregivers, employment, financial burden, quality of life

## Abstract

**Background:**

Little is known about how cancer impacts the employment status of patients’ family supporters, or about associations between patients’ health‐related quality of life, perceived financial burden, and supporters’ employment trajectory.

**Methods:**

We surveyed patients with early stage breast cancer reported to the Georgia and Los Angeles SEER registries in 2014–15, and their spouse/partner or other family supporters. Patients and supporters were asked about employment impacts of the patient’s cancer, and descriptive analyses of supporters’ employment trajectories were generated. We measured patients’ health‐related quality of life (HRQoL) using the PROMIS scale for global health. We measured patients’ perceived financial burden attributed to cancer by asking them two questions regarding (i) their financial status since their breast cancer diagnosis and (ii) how much it was impacted by their breast cancer and treatment. Associations between patients’ HRQoL, perceived financial burden, and supporters’ employment status were assessed using linear mixed model regression analyses.

**Results:**

In total, 2502 patients (68% response rate) and 1203 supporters (70% response rate) responded; 1057 paired patient‐supporter dyads were included. Similar proportions of spouse/partner and other family supporters reported missed work and lost employment due to patients’ cancer. After adjustment, lower HRQoL and an increased odds of perceived financial burden among patients were associated with changes in other family supporters’ employment (both *p* < 0.05), but not with changes in spouses’/partners’ employment. Lower HRQoL was also associated with changes in patients’ own employment among patients with both types of supporters (both *p* < 0.001). An increased odds of perceived financial burden among patients was associated with changes in patients’ employment only in those supported by other family members (*p* < 0.001).

**Conclusions:**

Both spouse/partner and other family supporters faced adverse employment outcomes due to patients’ cancer. This contributes to worse HRQoL and greater perception of financial burden among patients, especially those whose supporter is not a spouse/partner.

## Graphical abstract

We performed a large, population‐based survey study of diverse patients with early stage breast cancer and their spouses, partners, and other family supporters to understand how cancer impacts the employment status of patients’ spouse/partner and other family supporters, and about associations between patients’ health‐related quality of life, perceived financial burden, and their supporters’ employment trajectory. Both spouse/partner and other family supporters faced adverse employment outcomes due to patients’ cancer. This contributed to worse health‐related quality of life and greater perception of financial burden among patients, especially those whose supporter is not a spouse/partner.

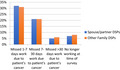

## 1 BACKGROUND

Working patients with cancer must make treatment decisions that often result in long‐term adverse impacts on their financial well‐being, employment trajectory, and health‐related quality of life (HRQoL).^1^, ^2^, ^3^, ^4^ Many patients are supported through their cancer diagnosis and treatment by spouses/partners or other family members who provide emotional support and caregiving while also balancing their own paid employment.^5^, ^6^, ^7^, ^8^, ^9^ In our prior work surveying spouses of women with early stage breast cancer, we found that spouses faced adverse employment and financial outcomes attributed to their wife’s cancer, and that spouses with greater perceived financial burden reported significantly lower emotional quality of life.^10^ When family supporters miss or stop work because of patients’ cancer, the financial consequences extend to patients and may be especially devastating in families that rely on these supporters as a major source of income. It therefore stands to reason that patients’ HRQoL and perceptions of their own financial burden due to their cancer—which significantly impacts HRQoL—may also be affected by the employment trajectory of their family supporters, though this has not been previously studied.

To address this gap, and to expand investigations to include non‐spouse family members who support patients through cancer diagnosis and treatment, we surveyed a large sample of diverse women with early stage breast cancer and their self‐identified key family supporter (i.e., spouses/partners or other family members). We report associations between patients’ HRQoL, their perceived financial burden, and supporters’ employment trajectories (including the trajectories of supporters who were not employed prior to patients’ diagnosis). We also describe missed and stopped work among the subset of supporters who were employed prior to patients’ diagnosis.

## 2 METHODS

### 2.1 Study population

As described previously,^11^ the Individualized Cancer Care (iCanCare) Study is a large, population‐based survey of women with breast cancer. We accrued 3930 women, ages 20–79, with stage 0–II breast cancer reported to the Georgia and Los Angeles County (LA) SEER registries in 2014–2015. Exclusion criteria included stage III/IV disease, tumors >5 cm, and inability to complete questionnaires in English or Spanish (*N* = 258). Patients were mailed surveys approximately 2 months after surgery (median time from diagnosis to survey completion = 7 months). We provided a $20 cash incentive and used a modified Dillman approach to encourage survey response.^12^, ^13^, ^14^ Survey responses were merged with SEER clinical data.

Patients were asked to list individuals who supported them in treatment decisions and identify the “most helpful” individual (key supporter)^11^; a separate survey packet was delivered to this individual. Eligible supporters were ≥age 21, able to read English or Spanish, and resided in the United States. The study was approved by the University of Michigan Institutional Review Board (IRB) and the state and institutional (Emory University and University of Southern California) IRBs of the SEER registries.

### 2.2 Family supporter employment outcomes

We asked all supporters about days of work missed due to the patient’s cancer diagnosis or treatment (None, <7, 7–14, 15–30, >30 days). We also assessed whether these supporters remained employed or stopped working completely following the patient’s cancer diagnosis by selecting supporters who were working for pay prior to the patient’s diagnosis and were not working for pay (and were not retired) at the time they completed the survey. We assessed supporters’ employment trajectory by comparing their self‐reported employment status prior to the patient's diagnosis and at survey completion. Employment trajectories were categorized as: (i) employed at diagnosis and at survey, (ii) employed at diagnosis and unemployed at survey, (iii) unemployed at diagnosis and at survey, or (iv) unemployed at diagnosis and employed at survey.

### 2.3 Additional family supporter variables

Supporters were asked to specify their relationship to the patient and were categorized into one of two supporter types: spouse/partner or other family member. Non‐family supporters were excluded from these analyses because we did not conceptualize an association between employment trajectories of non‐family supporters and patients’ HRQoL and financial burden. Given the sociodemographic differences between partnered and unpartnered patients we have found in our previous research,^11^, ^15^ and to better understand the employment impacts of patients’ cancer diagnoses and treatment on non‐partner family supporters as compared to partners, we stratified our analyses by supporter type (spouse/partner vs. other family member). Supporters also reported their age, race and ethnicity, educational attainment (high school or less, some college, or higher) and comorbid conditions (0, ≥1).

### 2.4 Patient independent variables

Because of expected co‐linearity between supporter and patient sociodemographic factors, only patients’ self‐reported marital status (married/partnered, not married/partnered), income (<$40,000, $40,000–$89,999, ≥$90,000), and relevant clinical factors were included in these analyses. Consistent with prior work,^16^ patients reported their comorbid conditions (0, ≥1) and receipt of chemotherapy (y/n), radiation therapy (y/n), and primary surgical treatment (lumpectomy, mastectomy). Breast cancer stage (0, I, II) was obtained from SEER. We assessed patients’ employment trajectory using the same method as for supporters.

### 2.5 Measures of patient HRQoL and perceived financial burden

We assessed two patient‐reported outcomes: (i) HRQoL and (ii) perceived financial burden due to cancer. HRQoL was measured using the PROMIS‐Preference (PROPr) score^17^ derived from the PROMIS scale for global health version 1.2.^18^ To aid in the interpretation of results, we created a standardized HRQoL score with mean = 0 and standard deviation = 1. A difference in 0.2 on the scale (0.2 standard deviation) is usually considered a small difference, 0.5 is considered a medium difference, and 0.8 is considered a large difference.^19^

Perceived financial burden was assessed by asking patients two questions: (i) are they worse off financially since their breast cancer diagnosis (Yes/No) and (ii) if Yes, how much of their financial burden is due to their breast cancer and treatment (5‐point Likert scale from “Not at all” to “Very much”). Patients who answered “Yes” they are worse off and that this is at least “Somewhat” due to breast cancer (versus “Not at all” or “A little”) were considered to have perceived financial burden due to their cancer.

### 2.6 Statistical analyses

First, we generated descriptive statistics of employment outcomes among supporters, stratified by supporter type (spouse/partner vs. other family member). Second, we examined HRQoL and financial burden among patients, stratified by their supporter type and controlling for supporter‐ and patient‐level characteristics including the employment trajectories of supporters and patients. Bivariate associations were evaluated using the chi‐squared tests. Multivariable regression models were estimated to predict adjusted HRQoL and financial burden among patients while controlling for supporter‐ and patient‐level covariates.

To reduce potential bias due to non‐response, weights were created using a logistic regression of supporter non‐response on demographic characteristics of the patients and used in multivariable analysis.^20^ Missingness was assessed for all variables. Any variables with more than 10% missing were imputed using multiple imputation methods and imputed values were used in all multivariable models. All statistical tests were two‐sided; *p*‐values < 0.05 were considered significant. Analyses were conducted with SAS 9.4 (Cary, NC).

## 3 RESULTS

Of 3672 eligible patients, 2502 completed surveys (68% response rate). Among 1713 eligible supporters, 1203 completed surveys (70% response rate). In total, 1057 paired patient‐supporter dyads were included in these analyses. Non‐family supporters were excluded from these analyses (*N* = 122). Missingness was less than 5% for all variables except income (missing = 14%), which was imputed using multiple imputation for multivariable models. Characteristics of patients and supporters are shown in Table 1. Most (81%) patients were married/partnered. Spouses/partners comprised 48% of supporters and other family comprised 52% of supporters. Spouse/partner supporters were more likely than other family supporters to be White, to be age 50–64, and to have some college education or more. Daughters comprised 46% of other family supporters (*N* = 249). Other family supporters were more likely than spouse/partner supporters to be Black or Latinx, to be age <50, and to have a high school education or less.

**TABLE 1:** Characteristics of patient and partner sample

Characteristic	Patients (*N* = 1057) No. (%)	Spouse/partner supporters (*N* = 512) No. (%)	Other family supporters (*N* = 545) No. (%)
Age, years			
<50	192 (19%)	97 (19%)	317 (59%)
50–64	470 (45%)	219 (43%)	138 (25%)
≥65	375 (36%)	192 (37%)	81 (15%)
Missing	20 (2%)	4 (1%)	9 (2%)
Race			
White	549 (52%)	354 (70%)	197 (37%)
Black	163 (15%)	55 (11%)	113 (21%)
Latinx	245 (23%)	62 (12%)	177 (33%)
Asian	87 (8%)	34 (7%)	44 (8%)
Other	13 (1%)	3 (1%)	8 (1%)
Missing	4 (<1%)	4 (1%)	6 (1%)
Education			
HS or less	333 (32%)	81 (16%)	130 (24%)
Some college or more	711 (68%)	429 (84%)	409 (75%)
Missing	13 (1%)	2 (<1%)	6 (1%)
Employment at time of patient's diagnosis			
Employed	515 (49%)	337 (66%)	318 (68%)
Unemployed	177 (17%)	170 (34%)	173 (32%)
Retired*	353 (34%)	N/A	N/A
Missing	12 (1%)	5 (1%)	12 (2%)
Employment trajectory			
Employed →Employed	392 (38%)	302 (60%)	318 (60%)
Employed →Unemployed	206 (20%)	34 (7%)	41 (8%)
Unemployed →Unemployed	422 (42%)	165 (33%)	153 (30%)
Unemployed →Employed	4 (<1%)	5 (1%)	19 (3%)
Missing	33 (3%)	6 (1%)	14 (3%)
Patient income		N/A	N/A
<$40,000	301 (33%)		
$40,000–$89,999	298 (33%)		
$90,000+	309 (34%)		
Missing	149 (14%)		
Patient marital status		N/A	N/A
Married/partnered	641 (81%)		
Not married/unpartnered	148 (19%)		
Comorbid conditions			
0	712 (67%)	386 (75%)	326 (60%)
1+	345 (33%)	126 (25%)	219 (40%)
Stage at diagnosis		N/A	N/A
0	160 (16%)		
I/II	865 (84%)		
Missing	32 (3%)		
Surgical procedure		N/A	N/A
Lumpectomy	639 (61%)		
Mastectomy	405 (39%)		
Missing	13 (1%)		
Receipt of radiation		N/A	N/A
No	520 (50%)		
Yes	516 (50%)		
Missing	21 (2%)		
Receipt of chemotherapy		N/A	N/A
No	712 (68%)		
Yes	330 (32%)		
Missing	15 (1%)		
Geographic site			
Los Angeles	542 (51%)	301 (59%)	214 (39%)
Georgia	515 (49%)	211 (41%)	331 (61%)

* Supporters were not asked about retirement status at the time of patients’ diagnosis.

### 3.1 Employment outcomes among supporters

Both spouse/partner and other family supporters reported adverse employment outcomes due to patients’ cancer. Sixty‐six percent of spouses/partners and 68% of other family supporters were employed at the time of the patient’s diagnosis. Among spouse/partners, 32% missed 1–7 days of work, 21% missed 7–30 days, and 5% missed >30 days. Among other family supporters, 31% missed 1–7 days of work, 20% missed 7–30 days, and 6% missed >30 days. Among supporters who were employed at the time of the patient’s diagnosis, 7% of spouses/partners and 8% of other family supporters were no longer employed at the time of survey completion (e.g., had an employment trajectory of employed at diagnosis and unemployed at survey).

### 3.2 Patient‐reported HRQoL and financial outcomes

#### 3.2.1 Health‐related quality of life

We present a standardized score for patient‐reported HRQoL with mean = 0, standard deviation = 1, and score range for our study sample of −3.7 to 1.7. Results of bivariable analyses with patients’ HRQoL are shown in Table 2. After multivariable adjustment for supporter‐ and patient‐related covariates, among patients with spouse/partner supporters, lower patient‐reported HRQoL was significantly associated with patient employment trajectory (difference in mean patient HRQoL score for employed→unemployed compared to employed→employed: −0.45; 95% CI −0.70, −0.20 and difference in mean patient HRQoL score for unemployed→unemployed compared to employed→employed: −0.28; 95% CI −0.47, −0.08; *p* < 0.001). Among patients with other family supporters, lower patient‐reported HRQoL was significantly associated with supporter employment trajectory (difference in mean patient HRQoL score for employed→unemployed compared to employed→employed: −0.06; 95% CI −0.42, −0.30 and difference in mean patient HRQoL score for unemployed→unemployed compared to employed→employed: −0.34; 95% CI −0.57, −0.11; *p* = 0.014) and patient employment trajectory (difference in mean patient HRQoL score for employed→unemployed compared to employed→employed: −0.51; 95% CI −0.74, −0.27 and difference in mean patient HRQoL score for unemployed→unemployed compared to employed→employed: −0.53; 95% CI −0.72, −0.34; *p* < 0.001) (Table 3).

**TABLE 2:** Bivariate analyses of patient health‐related quality of life, stratified by type of supporter

Characteristic	Spouse/partner supporter		Other family supporter	
	Mean patient HRQoL score	*p*	Mean patient HRQoL score	*p*
Supporter age, years		0.147		0.258
<50	0.01		−0.02	
50–64	0.20		0.08	
≥65	0.36		−0.07	
Supporter race		0.312		0.002
White	0.26		0.18	
Black	0.25		−0.16	
Latinx	0.20		0.06	
Asian	0.01		−0.18	
Other	−0.57		−0.20	
Supporter education		<0.001		0.004
HS or less	−0.09		−0.18	
Some college or more	0.26		0.04	
Supporter employment trajectory		<0.001		<0.001
Employed →Employed	0.23		0.12	
Employed →Unemployed	0.20		−0.14	
Unemployed →Unemployed	0.19		−0.23	
Patient marital status				0.235
Married/partnered			0.08	
Not married/unpartnered			−0.07	
Patient income		<0.001		<0.001
<$40,000	−0.34		−0.28	
$40,000–$89,999	0.14		0.12	
$90,000+	0.42		0.46	
Patient comorbid conditions		<0.001		<0.001
0	0.35		0.17	
1+	−0.24		−0.29	
Patient stage at diagnosis		0.178		0.701
0	0.35		0.00	
I/II	0.19		−0.01	
Patient surgical procedure		0.012		0.050
Lumpectomy	0.29		0.06	
Mastectomy	0.09		−0.12	
Patient receipt of radiation		<0.001		0.009
No	0.03		−0.15	
Yes	0.41		0.14	
Patient receipt of chemotherapy		<0.001		0.009
No	0.36		0.08	
Yes	−0.13		−0.19	
Patient employment trajectory		.242		0.020
Employed →Employed	0.38		0.35	
Employed →Unemployed	−0.09		−0.32	
Unemployed →Unemployed	0.17		−0.17	
Geographic site		0.948		<0.001
Los Angeles	0.21		−0.10	
Georgia	0.21		0.12	

Note:

To aid in the interpretation of results a standardized quality of life score is used, where mean = 0 and standard deviation = 1.

**TABLE 3:** Multivariable analyses of patient health‐related quality of life due to breast cancer, stratified by type of supporter

Characteristic	Spouse/partner supporter		Other family supporter	
	Difference in mean patient HRQoL score	*p*	Difference in mean patient HRQoL score	*p*
Supporter age (per 10 years)	0.15 (0.06, 0.25)	0.002	0.05 (−0.02, 0.11)	0.187
Supporter race		0.688		0.094
White	Ref		Ref	
Black	0.09 (−0.21, 0.40)		0.10 (−0.24, 0.26)	
Latinx	0.11 (−0.20, 0.41)		−0.01 (−0.32, 0.29)	
Asian	−0.12 (−0.48, 0.24)		0.39 (0.03, 0.75)	
Supporter education		0.124		0.032
HS or less	Ref		Ref	
Some college or more	0.24 (−0.07, 0.54)		0.25 (0.02, 0.49)	
Supporter employment trajectory		0.749		0.014
Employed →Employed	Ref		Ref	
Employed →Unemployed	−0.12 (−0.47, 0.22)		−0.06 (−0.42, 0.30)	
Unemployed →Unemployed	−0.06 (−0.28, 0.16)		−0.34 (−0.57, −0.11)	
Patient marital status				0.192
Married/partnered			Ref	
Not married/unpartnered			0.12 (−0.06, 0.30)	
Patient income		0.008		0.114
<$40,000	Ref		Ref	
$40,000–$89,999	0.32 (0.04, 0.60)		0.19 (−0.01, 0.38)	
$90,000+	0.46 (0.17, 0.75)		0.23 (−0.04, 0.50)	
Patient comorbid conditions		<0.001		<0.001
0	Ref		Ref	
1+	−0.46 (−0.65, −0.27)		−0.46 (−0.66, −0.27)	
Patient stage at diagnosis		0.962		0.465
0	Ref		Ref	
I/II	−0.01 (−0.27, 0.26)		0.01 (−0.16, 0.35)	
Patient surgical procedure		0.989		0.024
Lumpectomy	Ref		Ref	
Mastectomy	0.00 (−0.28, 0.27)		−0.29 (−0.55, −0.04)	
Patient receipt of radiation		0.077		0.643
No	Ref		Ref	
Yes	0.25 (−0.03, 0.52)		−0.06 (−0.31, 0.19)	
Patient receipt of chemotherapy		0.014		0.010
No	Ref		Ref	
Yes	−0.27 (−0.48, −0.06)		−0.28 (−0.49, −0.07)	
Patient employment trajectory		<0.001		<0.001
Employed →Employed	Ref		Ref	
Employed →Unemployed	−0.45 (−0.7, −0.2)		−0.51 (−0.74, −0.27)	
Unemployed →Unemployed	−0.28 (−0.47, −0.08)		−0.53 (−0.72, −0.34)	
Geographic site		0.781		0.019
Los Angeles	Ref		Ref	
Georgia	−0.03 (−0.23, 0.17)		0.27 (0.04, 0.50)	

Note:

Supporter race category “Other” and supporter employment trajectory category “Unemployed→Employed” not shown due to small N. To aid in the interpretation of results a standardized quality of life score is used, where mean = 0 and standard deviation = 1.

Other factors significantly associated with lower patient‐reported HRQoL among patients with spouse/partner supporters are shown in Table 3 and include greater supporter age, lower patient income, more patient comorbid conditions, and patient receipt of chemotherapy. Other factors significantly associated with lower patient‐reported HRQoL among patients with other family supporters are shown in Table 3 and include lower educational attainment among supporters, patient receipt of mastectomy versus lumpectomy, and patient receipt of chemotherapy (all *p* < 0.05).

#### 3.2.2 Financial burden

Perceived financial burden was endorsed by 38% of patients. Among these, 3% reported that their financial burden was “not at all” related to their cancer, 23% “a little,” 27% “somewhat,” 25% “quite a bit,” and 22% “very much.” Results of bivariable analyses of patients’ financial burden are shown in Table 4. After multivariable adjustment for supporter‐ and patient‐related covariates, among patients with spouse/partner supporters, an increased odds of patient‐reported financial burden was not significantly associated with patient or supporter employment trajectory. Among patients with other family supporters, an increased odds of patient‐reported financial burden was significantly associated with supporter employment trajectory (OR for employed→unemployed compared to employed→employed: 1.389; 95% CI 0.493, 3.911 and OR for unemployed→unemployed compared to employed→employed: 2.557; 95% CI 1.437, 4.55; *p* = 0.006), and patient employment trajectory for those patients who were employed at diagnosis and unemployed at the time of survey completion (OR for employed→unemployed compared to employed→employed: 1.974; 95% CI 0.954, 4.084; *p* < 0.001) (Table 5).

**TABLE 4:** Bivariate analyses of patient perceived financial burden due to breast cancer, stratified by type of supporter

Characteristic	Spouse/partner supporter *N* = 512		Other family supporter *N* = 545	
	Proportion with financial burden No. (%)	*p*	Proportion with financial burden No. (%)	*p*
Supporter age, years		0.004		0.434
<50	37 (39%)		113 (40%)	
50–64	84 (44%)		41 (33%)	
65–74	44 (25%)		32 (41%)	
Supporter race		0.913		0.031
White	114 (34%)		59 (31%)	
Black	20 (39%)		39 (41%)	
Latinx	19 (33%)		72 (45%)	
Asian	10 (29%)		14 (36%)	
Other	1 (33%)		5 (71%)	
Supporter education		0.673		0.130
HS or less	27 (36%)		52 (44%)	
Some college or more	138 (34%)		37 (37%)	
Supporter employment trajectory		0.069		0.030
Employed →Employed	111 (40%)		96 (33%)	
Employed →Unemployed	7 (30%)		15 (41%)	
Unemployed →Unemployed	45 (29%)		65 (46%)	
Patient income		<0.001		<0.001
<$40,000	42 (60%)		97 (49%)	
$40,000–$89,999	57 (40%)		50 (34%)	
$90,000+	55 (24%)		24 (24%)	
Patient marital status				0.620
Married/partnered			72 (37%)	
Not married/unpartnered			119 (40%)	
Patient comorbid conditions		0.575		0.413
0	119 (32%)		123 (40%)	
1+	47 (35%)		73 (39%)	
Patient stage at diagnosis		0.363		0.094
0	24 (30%)		36 (47%)	
I/II	140 (41%)		149 (37%)	
Patient surgical procedure		0.009		0.087
Lumpectomy	85 (31%)		110 (36%)	
Mastectomy	81 (43%)		79 (44%)	
Patient receipt of radiation		0.021		0.703
No	95 (39%)		94 (40%)	
Yes	70 (29%)		95 (38%)	
Patient receipt of chemotherapy		0.002		<0.001
No	101 (29%)		104 (31%)	
Yes	63 (44%)		83 (54%)	
Patient employment trajectory		0.005		<0.001
Employed →Employed	70 (33%)		67 (40%)	
Employed →Unemployed	40 (49%)		61 (60%)	
Unemployed →Unemployed	53 (29%)		56 (27%)	
Geographic site		0.002		0.571
Los Angeles	52 (26%)		113 (38%)	
Georgia	114 (39%)		78 (40%)	

**TABLE 5:** Multivariable analyses of patient financial burden due to breast cancer, stratified by type of supporter

Characteristic	Spouse/partner supporter		Other family supporter	
	Had financial burden (OR, 95% CI)	*p*	Had financial burden (OR, 95% CI)	*p*
Supporter age (per 10 years)	0.712 (0.54–0.94)	0.017	0.918 (0.754–1.118)	0.396
Supporter race		0.919		0.297
White	Ref		Ref	
Black	0.826 (0.341–2.001)		1.23 (0.598–2.531)	
Latinx	1.187 (0.467–3.014)		1.606 (0.711–3.626)	
Asian	0.826 (0.341–2.001)		2.616 (0.979–6.992)	
Supporter education		0.115		0.155
HS or less	Ref		Ref	
Some college or higher	1.699 (0.878–3.286)		0.634 (0.339–1.189)	
Supporter employment trajectory		0.157		0.006
Employed →Employed	Ref		Ref	
Employed →Unemployed	0.5 (0.166–1.509)		1.389 (0.493–3.911)	
Unemployed →Unemployed	0.553 (0.282–1.084)		2.557 (1.437–4.55)	
Patient marital status	N/A	N/A		0.391
Married/partnered			Ref	
Not married/unpartnered			0.791 (0.463–1.353)	
Patient income		<0.001		0.042
<$40,000	Ref		Ref	
$40,000–$89,999	0.339 (0.171–0.672)		0.638 (0.359–1.134)	
$90,000+	0.141 (0.067–0.300)		0.363 (0.161–0.817)	
Patient comorbid conditions		0.285		0.942
0	Ref		Ref	
1+	1.344 (0.781–2.314)		0.983 (0.579–1.668)	
Patient stage at diagnosis		0.973		
0	Ref		Ref	<0.001
I/II	0.989 (0.510–1.917)		0.277 (0.136–0.564)	
Patient surgical procedure		0.117		0.057
Lumpectomy	Ref		Ref	
Mastectomy	1.755 (0.852–3.617)		1.864 (0.98–3.546)	
Patient receipt of radiation		0.636		0.006
No	Ref		Ref	
Yes	1.193 (0.574–2.477)		2.508 (1.311–4.796)	
Patient receipt of chemotherapy		0.047		<0.001
No	Ref		Ref	
Yes	1.778 (1.007–3.140)		3.388 (1.911–6.006)	
Patient employment trajectory		0.064		<0.001
Employed →Employed	Ref		Ref	
Employed →Unemployed	1.969 (1.032–3.755)		1.974 (0.954–4.084)	
Unemployed →Unemployed	0.972 (0.537–1.759)		0.284 (0.155–0.520)	
Geographic site		0.005		0.016
Los Angeles	Ref		Ref	
Georgia	2.455 (1.307–4.611)		2.279 (1.170–4.436)	

Note:

Supporter race category “Other” and supporter employment trajectory category “Unemployed→Employed” not shown due to small N.

Other factors significantly associated with an increased odds of patient‐reported financial burden among patients with spouse/partner supporters are shown in Table 5 and include lower supporter age, patient income <$40,000, and patient receipt of chemotherapy. Other factors significantly associated with an increased odds of patient‐reported financial burden among patients with other family supporters are shown in Table 5 and include patient income <$40,000, patient receipt of radiation, and patient receipt of chemotherapy (all *p* < 0.05).

The interaction between income and employment trajectory was assessed and was not significantly associated with HRQoL or financial burden.

## 4 DISCUSSION

Our large, population‐based survey study of diverse women with breast cancer is unique because, in addition to surveying the patients, we also surveyed their self‐identified spouse/partner and other family supporters to elicit their own employment outcomes. We found that spouse/partner and other family supporters reported both missed work due to patients’ cancer and lost employment. Notably, other family supporters reported missed work and lost employment in proportions similar to those of spouse/partner supporters. Moreover, we found that patients commonly perceived financial burden that they attributed to their cancer. Lower patient‐reported HRQoL and an increased odds of patient‐reported financial burden were associated with changes in other family supporters’ employment trajectories (from employed to unemployed or unemployed to unemployed), but not with changes in spouses/partners’ employment trajectories.

According to the United States Bureau of Labor Statistics, the national unemployment rate among adults in November 2015 was 5%.^21^ In comparison, 7% of the spouse/partner supporters and 8% of other family supporters in our study who were employed at the time of patients’ diagnosis were unemployed at the time of survey completion. While we do not know if this loss of employment among supporters was voluntary or involuntary, any employment loss represents a resultant loss of income. Thus, there is a need to better understand the relationship between employment trajectories and financial burden in families affected by cancer, as employment outcomes and financial burden are naturally intertwined.

Overall patient‐reported HRQoL in this study was comparable to other published results in patients with breast cancer.^22^ In addition to our finding that lower patient HRQoL was associated with changes in other family supporter's employment trajectories, we also found that lower HRQOL among patients was associated with changes in patients’ own employment trajectory. Among patients with spouse/partner supporters, we found the lowest mean patient‐reported HRQoL scores in patients who were employed at the time of their cancer diagnosis and unemployed at the time of survey completion. Among patients with other family supporters, we found very similar mean patient‐reported HRQoL scores in patients who were either employed or unemployed at the time of diagnosis and unemployed at the time of survey completion. In comparison to the very small effect size associated with supporters’ employment trajectories, the effect sizes associated with patients’ employment trajectories suggest that patients’ own employment trajectory is the primary driver of their perceptions of their own HRQoL.

Moreover, we found that an increased odds of patient‐reported financial burden was associated with changes in other family supporters’ employment trajectories, but not with changes in spouses’ employment trajectories. Additionally, an increased odds of patient‐reported financial burden was associated with changes in patients’ employment trajectory only in those with other family supporters. Prior work has found spouses’ perceived financial burden to be associated with changes in patients’ employment trajectory (greatest perceived financial burden when patient goes from employed to unemployed).^10^ A possible explanation for our findings is that patients who have another family member as their key supporter differ from patients who have a spouse/partner supporter in ways we were not able to measure in this study. Notably, 44% of these patients in our study also have a spouse or partner who they did *not* report as their key support person. Perhaps for patients with another family supporter, who in our study were more likely to be under age 50, Black or Latina, and have a lower level of educational attainment than patients with a spouse/partner supporter, the impact of other family supporters’ employment is more noticeable because of unique family arrangements, including financial arrangements, that may be in place. Taken together, our results suggest that the employment trajectories of patients and their supporters—both spouses/partners and other family supporters—are intertwined and impact important patient‐ and family‐centered outcomes. Further research is warranted to elucidate the specific financial and employment needs of patients who are supported by other family members and not by a spouse or partner.

Though our study included a large, diverse sample of patients and supporters recruited from urban and rural settings, our findings are limited to patients with non‐metastatic cancer recruited from Georgia and Los Angeles and may not be generalizable to all patients. As with all survey studies, nonresponse bias is possible. When compared with patients whose supporters responded to the survey, patients whose supporters did not respond were more likely to be Black and to have lower educational attainment. The inclusion of a non‐cancer control group was beyond the scope of this study. However, we attempted to mitigate this by asking whether employment outcomes and financial status were perceived *as a result of* patients’ cancer.

Based on our findings and the limited extant literature describing employment outcomes among spouses and family caregivers of cancer patients, it is important for clinicians to recognize that both patients and their family supporters are at risk for missed work, job loss, and financial burden after a cancer diagnosis. It is also important for clinicians to understand that even some patients who are married are primarily supported by another family member, and that these other family supporters can face adverse employment outcomes as a result of the patient’s cancer that negatively impact patients’ own HRQoL and financial burden. It has been suggested that clinicians should start conversations with patients about financial and employment concerns soon after diagnosis and continue these conversations throughout cancer care.^23^ Policies at the local and national levels, including expansion and provision of financial resources to support patients through cancer diagnosis and treatment,^24^ and employer‐based accommodations such as paid sick leave and flexible scheduling,^25^, ^26^ may help mitigate adverse financial and employment effects. Our findings suggest that in order to best support patients and families, spouse/partner and other family supporters should be included in these important conversations and policy considerations.

## CONFLICT OF INTEREST

None.

## AUTHOR CONTRIBUTIONS

Christine Veenstra: conceptualization, methodology, investigation, writing original draft, writing review and editing, and visualization. Thomas Braun: conceptualization, methodology, investigation, and writing review and editing. Paul Abrahamse: methodology, software, formal analysis, investigation, writing original draft, writing review and editing, and visualization. Daniela Wittmann: conceptualization, methodology, investigation, and writing review and editing. Sarah Hawley: conceptualization, methodology, investigation, writing original draft, writing review and editing, and visualization.

## Data Availability

The data that support the findings of this study are available upon request from the corresponding author. These data are not publicly available due to privacy or ethical restrictions.
